# The complete chloroplast genome sequence of *Kadsura heteroclita*

**DOI:** 10.1080/23802359.2020.1768963

**Published:** 2020-05-27

**Authors:** Qinghua Wang, Huangyijun Wang, Yupin Fu, Yunqin Li, Xiaolong Yuan, Yi Wang

**Affiliations:** a Laboratory of Forest Plant Cultivation and Utilization, Yunnan Academy of Forestry and Grassland Science, Kunming, China; b College of Forestry, Fujian Agriculture and Forestry University, Fuzhou, China

**Keywords:** *Kadsura heteroclita*, chloroplast, Illumina sequencing, phylogenetic analysis

## Abstract

The first complete chloroplast genome (cpDNA) sequence of *Kadsura heteroclita* was determined from Illumina HiSeq pair-end sequencing data in this study. The cpDNA is 153,289 bp in length, contains a large single copy region (LSC) of 85,774 bp and a small single copy region (SSC) of 18,201 bp, which were separated by a pair of inverted repeats (IR) regions of 24,657 bp. The genome contains 129 genes, including 84 protein-coding genes, eight ribosomal RNA genes, and 37 transfer RNA genes. Further phylogenomic analysis showed that *K. heteroclita and K. interior* clustered in a clade in Schisandraceae family.

*Kadsura heteroclita* is the species of the genus *Kadsura* within the family Schisandraceae. It is a climbing species distributed in the southwest of China (Yang et al. 1992). The stems and roots of *K. heteroclita* are known as ‘Ji-Xue-Teng’, which have been used in the treatment of rheumatism arthritis, menstrual irregularities, blood deficiencies, and other feminine disorders (Wang et al. 2006). The extract of the stems from *K. heteroclita* also showed anti-HIV activity (Pu et al. 2008) and antioxidant and cytotoxic activities (Cao et al. 2020). *Kadsura heteroclita* has a huge medicinal value. However, there have been no genomic studies on *K. heteroclita*.

Herein, we reported and characterized the complete *K. heteroclita* plastid genome. The GenBank accession number is MN823698. One *K. heteroclita* individual (specimen number: 2020014) was collected from Kunming, Yunnan Province of China (25°8’13” N, 102°35’18” E). The specimen is stored at Yunnan Academy of Forestry Herbarium, Kunming, China, and the accession number is WQH003. DNA was extracted from its fresh leaves using DNA Plantzol Reagent (Invitrogen, Carlsbad, CA, USA).

Paired-end reads were sequenced using Illumina HiSeq system (Illumina, San Diego, CA). In total, about 22.3 million high-quality clean reads were generated with adaptors trimmed. Aligning, assembly, and annotation were conducted by CLC de novo assembler (CLC Bio, Aarhus, Denmark), BLAST, GeSeq (Tillich et al. 2017), and GENEIOUS v 11.0.5 (Biomatters Ltd, Auckland, New Zealand). To confirm the phylogenetic position of *K. heteroclita*, the other eight species of *Schisandraceae* family from NCBI were aligned using MAFFT v.7 (Katoh and Standley 2013). The Auto algorithm in the MAFFT alignment software was used to align the eleven complete genome sequences and the G-INS-i algorithm was used to align the partial complex sequences. The maximum likelihood (ML) bootstrap analysis was conducted using RAxML (Stamatakis 2006); bootstrap probability values were calculated from 1000 replicates. *Nuphar advena* (DQ354691) and *Nuphar longifolia* (MH050795) were served as the out-group.

The complete *K. heteroclita* plastid genome is a circular DNA molecule with the length of 153,289 bp, contains a large single copy region (LSC) of 85,774 bp and a small single copy region (SSC) of 18,201 bp, which were separated by a pair of inverted repeats (IR) regions of 24,657 bp. The overall GC content of the whole genome is 39.6%, and the corresponding values of the LSC, SSC, and IR regions are 38.8%, 34.9%, and 42.9%, respectively. The plastid genome contained 129 genes, including 84 protein-coding genes, eight ribosomal RNA genes, and 37 transfer RNA genes. Phylogenetic analysis showed that *K. heteroclita* and *K. interior* clustered in a clade in *Schisandraceae* family (Figure 1). The determination of the complete plastid genome sequences provided new molecular data to illuminate the *Schisandraceae* family evolution.

**Figure 1.**
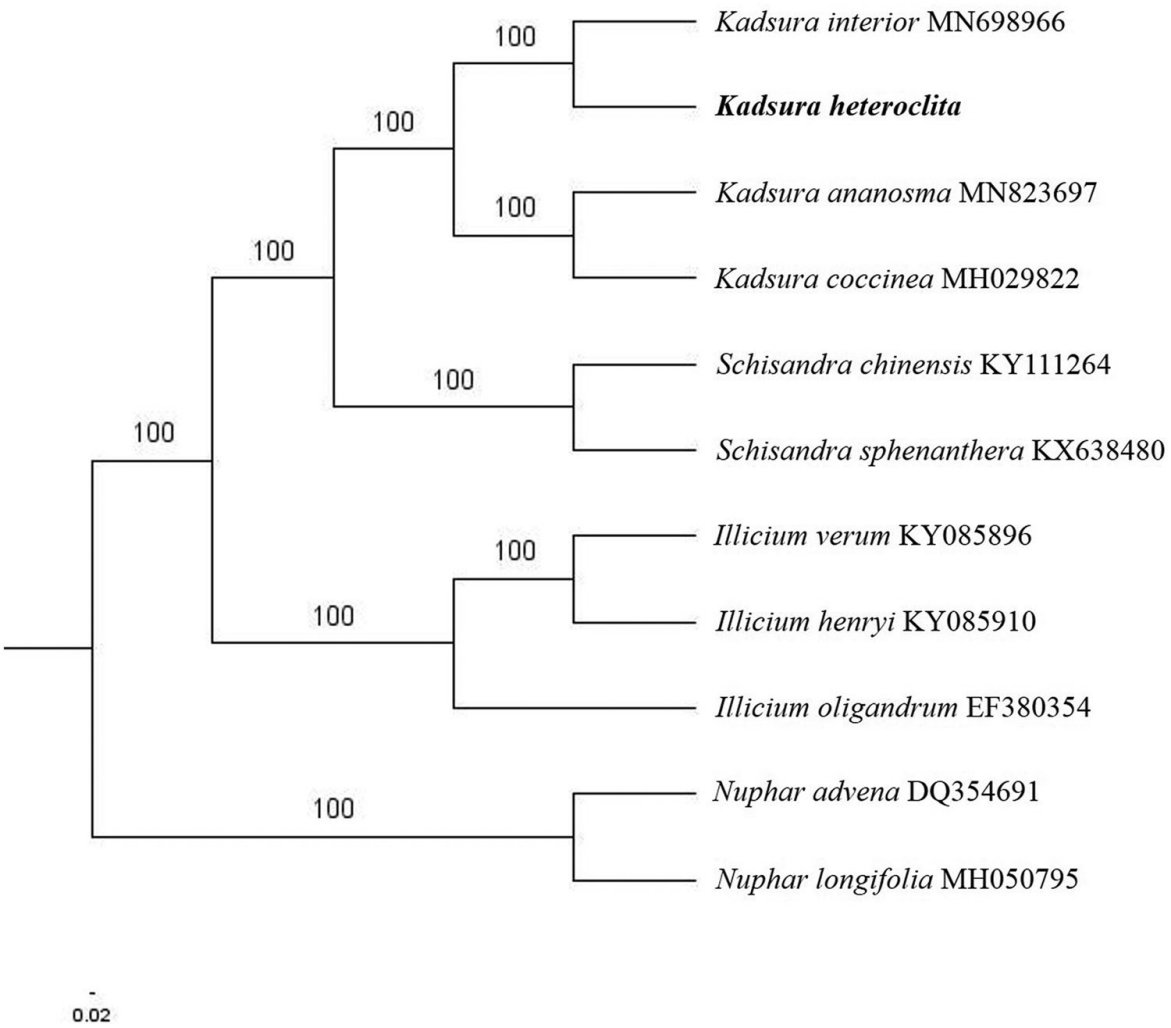
The maximum-likelihood tree based on the nine chloroplast genomes of *Schisandraceae* family. The bootstrap value based on 1000 replicates is shown on each node.

## Data Availability

The data that support the findings of this study are openly available in NCBI GenBank database at (https://www.ncbi.nlm.nih.gov) with the accession number is MN823698, which permits unrestricted use, distribution, and reproduction in any medium, provided the original work is properly cited.
